# Building resilient health-care supply chains to manage pandemics in low- and middle-income countries

**DOI:** 10.2471/BLT.21.287177

**Published:** 2022-01-10

**Authors:** Genevie Fernandes, Ines Hassan, Devi Sridhar

**Affiliations:** a Global Health Governance Programme, Usher Institute, University of Edinburgh, Teviot Place, Edinburgh EH8 9AG, Scotland.

In September 2021, representatives of the pharmaceutical industry projected that by January 2022 enough vaccines for coronavirus disease 2019 (COVID-19) will have been produced to vaccinate every adult in the world.^1^ However, after 5.48 million deaths globally up to 8 January 2022, access to this life-saving public good has arguably been too slow. Critically, vaccine access remains uneven today, with countries in Africa lagging severely behind in the number of doses administered to their populations.^2^ Manufacturers also did not foresee the need for booster doses, the vaccination of children and the wastage of vaccines. While there are multiple barriers contributing to this gap, including hoarding of vaccine supplies by high-income countries and the public’s hesitancy or refusal to vaccinate, it is evident that production capacity and supply-chain robustness remain important determinants of vaccine availability.^3^ The pandemic has also put unprecedented strain on the supply of other essential health commodities, including personal protective equipment, diagnostic tests and treatments.^4^^,^^5^ Supply problems have impacted the response to COVID-19 and disrupted essential health-care services particularly for child immunization, family planning, mental health disorders and cancer.^6^

Many scientists predict that severe acute respiratory syndrome coronavirus 2 (SARS-CoV-2) may become an endemic virus in the future. We are already witnessing repeat surges in COVID-19 cases involving new variants of concern, which are going to be especially detrimental in low- and middle-income countries where access to effective treatments and vaccines remains poor. Moreover, COVID-19 will be more difficult to manage if or when surges of infection coincide with other regional or global disasters, such as other infectious disease outbreaks, flooding or forest fires. To prepare for these challenges, the root causes of production and supply‑chain issues that are still delaying access to vital pandemic tools must be addressed urgently. 

## What went wrong?

The pandemic has exposed countries’ reliance on highly globalized supply chains. The production of active pharmaceutical ingredients, finished vaccines and essential medical supplies is concentrated in a limited number of countries, delaying global supply amidst surges in demand.^3^^,^^5^ Even locally manufactured products rely on materials produced in countries such as China and India.^7^ Intellectual property laws have led to dependency on a small number of vaccine manufacturers, many of which are located in high-income countries.^3^ Furthermore, staff absence due to illness or quarantine has shut down or slowed manufacturing. Rapid changes in cargo operations, airport and seaport closures and restrictions on population movements have made the transport of goods more expensive and unpredictable.^7^ Purchases of more vaccine doses than needed, together with export restrictions on COVID-19-related commodities by certain countries, has triggered shortages and created inequities in other countries’ access to vaccines.^4^ More importantly, despite repeated calls from the World Health Organization (WHO), there has been no coordinated, global strategy to defeat the pandemic, resulting in inadequate sharing of essential global public goods by higher-income countries.

## What went well?

Some low- and middle-income countries have prepared, adapted and responded rapidly to the supply-chain issues during the pandemic. Forecasting a rise in demand for medical oxygen right at the start of the pandemic, the government taskforce in the Indian state of Kerala increased existing production, approved new manufacturing plants, diverted industrial oxygen cylinders for medical use and increased the number of hospital beds with oxygen capacity.^8^ Kerala managed surplus oxygen supply during the second wave of the pandemic, while other states of India struggled with acute shortages that led to preventable deaths. In western Kenya, a push-based supply strategy with electronic record monitoring was designed to supply essential medicines to patients while minimizing the risk of exposure to COVID-19.^9^ Decentralized warehouses were set up in peripheral health facilities and essential medicines were delivered to patients at drop-off points in the community or at their homes via collaboration with local authorities. Public–private partnerships in Nigeria and Uganda have resulted in the production of low-cost ventilators by automobile companies, highlighting an innovative approach of diversifying the supply chain for this vital commodity.^10^ Nigeria has developed a low-cost and rapid COVID-19 diagnostic test which can be mass-produced in the country, thereby reducing costs and reliance on international supplies.^11^

## The way forward

Despite these encouraging initiatives, intellectual property rights on COVID-19 vaccines are proving to be a barrier to expanding regional manufacturing and freeing up unused capacity in several low- and middle-income countries.^3^ In December 2021, the World Health Assembly adopted the decision to start working on “an international instrument to strengthen pandemic prevention, preparedness and response”.^12^ This international treaty could potentially use emergency waivers on intellectual property rights and licenses to allow the manufacturing of COVID-19 vaccines and essential medical goods in low- and middle-income countries. These measures would enable regional hubs to be established, more manufacturers to be commissioned and more doses of vaccine to be created. The treaty could include provisions to tackle export bans on essential medical products through diversifying supply chains and could also promote knowledge sharing and technology transfer for safe, standardized and speedy production. 

While such an international agreement is urgently needed, obtaining the approval and consensus of WHO Member States will take time. Meanwhile, low- and middle-income countries can start building their capacity to produce essential equipment and medicines that do not have any intellectual property-related restrictions. Until countries’ access to vaccines is secured, tools that protect and treat populations, such as personal protective equipment, testing kits and treatments, can be locally produced with government funding or via public–private partnerships. Throughout the pandemic, many African countries have been reliant on imported personal protective equipment that is often subject to supply disruptions and too costly for their limited procurement budgets. The speedy establishment of new personal protective equipment manufacturers during the pandemic, such as the O-Care medical face mask (Transgreen Nigeria Ltd, Lagos, Nigeria) has demonstrated the ease with which these tools can be produced locally by private organizations with government support. 

Low- and middle-income country governments can also use subsidies and other mechanisms to create incentives for existing local manufacturers of medical products to build excess capacity into the supply chain in the form of emergency stockpiles, diversified suppliers (including non-traditional sources) and increased manufacturing capacity. The surplus supplies may be used to facilitate trade negotiations as new treatments and vaccines emerge in advanced economies. Better use should be made of regional mechanisms, such as the African Continental Free Trade Area agreement, to ease existing export and import controls on essential medicines and to improve access to key supplies. 

In the mid- to long-term, high-income countries need to work with low- and middle-income countries to invest in building regional manufacturing hubs that can produce adequate supplies of complex vaccines, antiviral drugs, drugs derived from natural sources, diagnostic tests and medical devices. Such partnerships will help countries prepare for future COVID-19 surges or other infectious disease outbreaks while offering the incentives of health security and efficient flow of trade for high-income countries. One pharmaceutical company (BioNTech SE, Mainz, Germany) shared plans in May 2021 to build an mRNA vaccine manufacturing facility that will supply the South-East Asia Region with vaccines beyond the COVID-19 pandemic. Additionally, there are at least 12 manufacturing facilities across Africa that are producing or have agreed to produce the drug substance or final formulations of various COVID-19 vaccines. 

These partnerships do not go far enough, however. Regional facilities should comprise multistage processing from bulk production to the fill and finish stages and should include facilities that produce materials essential for production, such as single-use bioreactors and the necessary buffer solutions, reagents and salts. Manufacturing facilities should be able to produce enough supplies for their region. Furthermore, to work effectively, the initiative will require large-scale investment; collaboration among different organizations; skills and capacity-building; technology transfer; transparency in the entire supply-chain process for internal and external stakeholders; and potentially some waivers to intellectual property rights. 

In the long-term, investments are needed in subnational, national and regional surveillance systems for infectious disease outbreaks, including genomic surveillance of pathogens. These investments will enable low- and middle-income countries to better forecast and monitor new variants of SARS-CoV-2 and other pathogens and to issue timely and targeted responses to reduce the risk of sudden increases in demand for essential supplies and vaccines. Advanced analytic techniques can then be used to combine epidemiological and stock-related data to forecast the demand for these essential health goods. Such predictive forecasting will ensure a proactive health-care supply chain that anticipates any potential shifts in demand and consumption. Such systems will require large-scale investment, capacity- and skills-building of regional and local teams, and cooperation between the public and private sector as well as between high-income and low- and middle-income countries. Transparency in the process will also be needed to enable the transfer of clear and timely information and to build partnerships. 

Sizeable financial commitments from high-income countries and global health donors as well as domestic investments will be essential for implementing the above recommendations, along with improved governance. WHO’s proposed international treaty for pandemic prevention, preparedness and response shows promise for addressing global health governance issues. Government teams at central, provincial and district levels will have to be strengthened, incorporating genuine incentives for transparency and accountability in supply chains to ensure equitable distribution of essential health goods and avoid any wastage. Improved coordination between those working in the public, private and community sectors will be vital in ensuring that essential medical goods including vaccines reach the required users. Many high-income countries are already discussing the post-pandemic future. Yet without pledging significant changes in global pandemic preparedness, prioritizing the needs of high-income countries and low- and middle-income countries alike, the mistakes of the current pandemic will be repeated in the next global outbreak.
